# Overexpression of BZW1 is an independent poor prognosis marker and its down-regulation suppresses lung adenocarcinoma metastasis

**DOI:** 10.1038/s41598-019-50874-x

**Published:** 2019-10-10

**Authors:** Jean Chiou, Yu-Chan Chang, Yi-Hua Jan, Hsing-Fang Tsai, Chih-Jen Yang, Ming-Shyan Huang, Yung-Luen Yu, Michael Hsiao

**Affiliations:** 1 Genomics Research Center, Academia Sinica, Taipei, Taiwan; 2 Department of Internal Medicine, Kaohsiung Municipal Ta-Tung Hospital, Kaohsiung Medical University Hospital, Kaohsiung Medical University, Kaohsiung, Taiwan; 3 Department of Internal Medicine, E-DA Cancer Hospital, School of Medicine, I-Shou University, Kaohsiung, Taiwan; 4 The PhD. Program for Cancer Biology and Drug Discovery, China Medical University and Academia Sinica, Taichung, Taiwan; 5 Graduate Institute of Biomedical Science, China Medical University, Taichung, Taiwan; 6 Center for Molecular Medicine, China Medical University Hospital, Taichung, Taiwan; 7 Department of Biotechnology, Asia University, Taichung, Taiwan; 8 Department of Biochemistry, College of Medicine, Kaohsiung Medical University, Kaohsiung, Taiwan

**Keywords:** Non-small-cell lung cancer, Metastasis

## Abstract

The basic leucine zipper and the W2 domain-containing protein 1 (BZW1) plays a key role in the cell cycle and transcriptionally control the histone H4 gene during G1/S phase. Since cellular proliferation rates are frequently dysregulated in human cancers, we identified the characteristics of BZW1 in cancer cells and analyzed its prognostic value in lung cancer patients. By searching public databases, we found that high BZW1 expression was significantly correlated with poor survival rate in non-small cell lung cancer (NSCLC), especially in lung adenocarcinoma. Similar trends were also shown in an array comprising NSCLC patient tissue. Knockdown of BZW1 inhibited cell metastatic ability, but did not affect the cell proliferation rate of NSCLC cells. From transcriptomics data mining, we found that coordination between BZW1 and EGFR overexpression was correlated with a worse outcome for lung cancer patients. In summary, BZW1 expression serves as an independent prognostic factor of NSCLC, especially in lung adenocarcinoma. Overexpression of BZW1 in lung cancer cells revealed a novel pathway underlying the induction of lung cancer metastasis.

## Introduction

Lung cancer is the major cause of cancer death, and non-small cell lung cancer (NSCLC) occupied eighty percent of all cases worldwide^1^. Metastasis is a major cause of treatment failure and death for patients with NSCLC despite that chemotherapy and other standard guidelines have been applied in cancer treatment. The histological subtypes of lung cancer include adenocarcinoma, squamous cell carcinoma and large cell carcinoma^2^. Recently reports showed various subtypes and genetic alterations involved in the aggressive progression of lung cancer, but the precise mechanisms remain unclear. Although several biomarkers in clinical use have been published, none are suitable for further study in terms of the types of sensitivity and/or specificity. Herein, we have identified an important molecule involved in a signaling pathway that depends on its transcriptional activity and protein structure in lung cancer tumorigenesis.

The Basic Leucine Zipper and W2 domains 1 (BZW1, BZAP45) are members of the bZIP superfamily of transcription factors. The bZIP domain contains several proteins, including the ATF family, JUN, CREB and NRF2, that are important transcription factors^3^. Both BZW1 and the paralog gene BZW2 encode a 45 kDa protein containing a C-terminal nucleotide (ATP/GTP) binding domain and an N-terminal bZIP domain^4^. A leucine zipper was designated as a protein-protein interaction motif, while the basic region was determined to be in charge of DNA binding. Interactions between bZIP proteins and their transcription factors are myriad, complicated and play prominent roles in cancer development^5,6^. Human BZW1 can activate the transcription of the histone H4 gene and serve as a coregulator of other transcription factors to control the cell cycle^4^. In addition, as a proliferation inducer, BZW1 promotes salivary mucoepidermoid carcinoma cell growth^7^. Other functions of BZW1 in cancer are still unknown.

Here, we demonstrate that overexpression of BZW1 corresponds to a poor survival rate in patients with lung cancer, especially for those with the adenocarcinoma subtype^8^. Furthermore, knockdown of BZW1 expression inhibited the migration abilities of lung cancer cells. We also performed pathological analysis on a tissue array via immunohistochemistry staining. The results revealed that overexpression of BZW1 correlates with not only poor survival but also recurrence. Taken together, our data adumbrate that BZW1 could be a new diagnostic marker and even a promising therapeutic target for combating malignant lung cancer.

## Materials and Methods

### Case selection

We examined the demographic features of 111 patients with NSCLC between 1991 and 2007 were included in this study at the Kaohsiung medical university Hospital of Taiwan. Patients who received preoperative radiation therapy or chemotherapy were excluded. The study was officially approved by the Institutional Review Boards of Kaohsiung Medical University Hospital of Taiwan (KMUH-IRB-E(I)-20160099). The entity of the study is retrospective and only archival surgically removed tumor paraffin block samples were used, there was no registration number in the ClinicalTrials.gov website. Requirement for informed consent was waived by the Institutional Review Boards of Kaohsiung Medical University Hospital of Taiwan. Clinical information and pathology data were collected as described^9^. Overall survival (OS) and diseases-free survival (DFS) were defined as the interval from surgery to death caused by the non-small cell lung cancer and recurrence or distant metastasis, respectively.

### Immunohistochemistry analysis

We stained tissue slides with polyclonal rabbit anti-human BZW1 antibody (1:100, Cat# ab85090, Abcam, Cambridge, UK) using an automated immunostainer (Ventana Discovery XT, Ventana Med. Systems, AZ, USA). All procedures were performed by previously described^9,10^. Each sample was graded as 0, 1, 2 or 3 (from weak to strong) based on the staining intensity by two independent pathologists.

### Cell culture and materials

The human lung adenocarcinoma (ADC) cell lines, H928, H1355, A549, and human lung large cancer cell line, H1299 were purchased from ATCC. CL1-0 and CL1-5 were kind gifts from Dr. Pan-Chyr Yang’s Lab^11^. All cells were incubated under the procedure as previous described^9^. For establishing stable cell lines, the pGIPZ lentiviral shRNA system (Thermo, MA, USA) was used with the BZW1 sequence. Lentivirus was used to infect CL1-5 cells for two days. We selected stable clones with 1 μg/ml puromycin (Sigma-Aldrich, MO, USA) for 14 days. The methods followed the procedure in our lab^10^.

### RNA extraction and RT-PCR analysis

We lysed cells by TRIzol reagent (Invitrogen, CA, USA), and total RNA was extracted by following the manufacturer’s procedures. The quality and amount of RNA was measured by Nanodrop spectrophotometer (Thermo, MA, USA)^10^. Reverse transcription-PCR (RT-PCR) was executed using a SuperScript III kit (Invitrogen, CA, USA) according to the manufacturer’s procedures. The expression levels of target genes were normalized to 26S ribosomal protein, which was used as an internal control. The primer sequences were as follows: BZW1-Forward: ACTGGTGTTCTTCTGGCTAA; BZW1-Reverse: GTGCTCATTACACTTGACCA; 26S-Forward: CCGTGCCTCCAAGATGACAAAG, and 26S-Reverse: ACTCAGCTCCTTACATGGGCTT.

### Cell migration and invasion assay

Polycarbonate filters (GE, Boston, MA, USA) were coated with 50 μL of Matrigel on the upper side (only invasion) and human fibronectin on the lower side. Medium containing 10% FBS was added to each lower well. Cells were mixed in 0% FBS medium and loaded into each upper well. After a suitable amount of time, the cells were fixed in methanol for 10 minutes then stained and counted on the lower side of the membrane under a light microscope (400×, 8 random fields from each well). All experiments were conducted in triplicate.

### Cell viability measurements

We used the MTT reagent (Trevigen, Gaithersburg, MD, USA) according to the manufacturer’s instructions^10^ to examine cell viability. 2000 cells/well were seeded a 96-well plate. At 24 hours postseeding, the cells viability was measured by MTT for 24 hrs, 48 hrs or 72 hrs, respectively. We further measured the optical density at 570 nm with a microplate reader (Spectral Max250; Molecular Devices, CA, USA).

### Statistical analysis

Statistics analysis was performed using SPSS for Windows (Version 17.0, Chicago, Illinois, USA). We analyzed associations between clinicopathological variables and BZW1 IHC expression by Pearson’s chi-square test. Comparing the BZW1 IHC expression in cancerous tissue to that in the corresponding normal lung tissue, we used a paired t-test. Overall survival (OS) and diseases-free survival (DFS) rates were evaluated with Kaplan-Meier method. Several clinicopathological variables, like tumor stage, lymph node stage and metastasis, were considered for univariate and multivariate analyses using Log-rank test and Cox proportional hazards regression analysis with and without adjustment for the BZW1 IHC expression level. For all of the analyses, a value of p < 0.05 was considered statistically significant.

## Results

### BZW1 is overexpressed in multiple cancer patient cohorts

**Figure 1:**
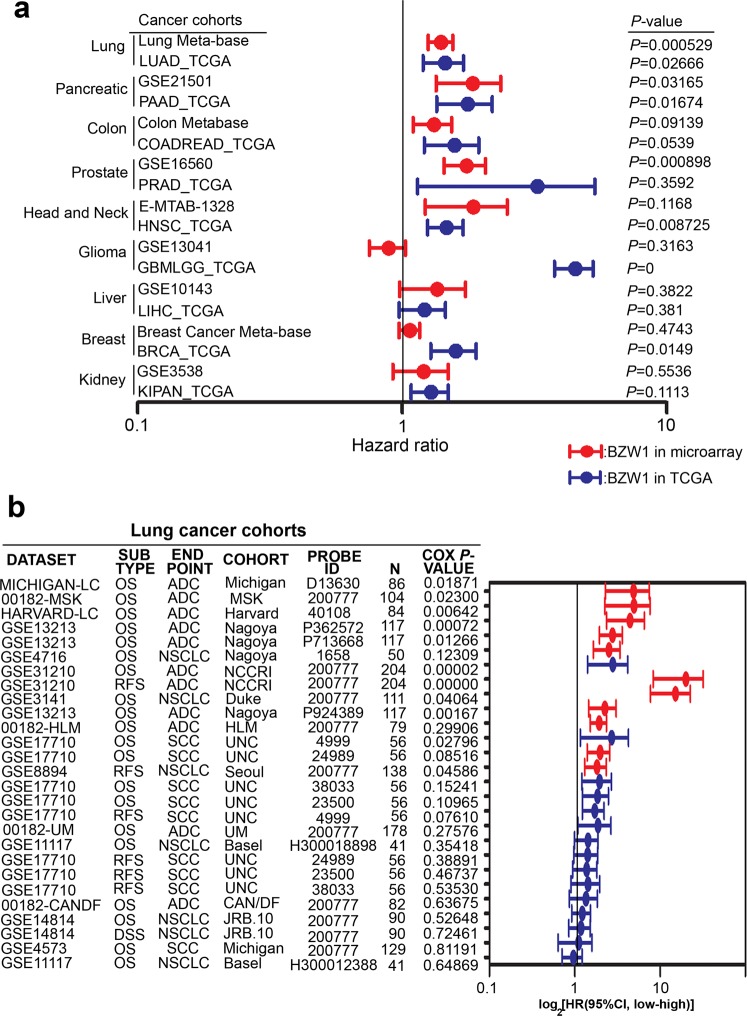
BZW1 as a poor prognostic factor in multiple carcinogenesis. (**a**) A meta-analysis for BZW1 gene against clinical cohorts with multiple cancer includes microarray and TCGA cohort by using Survexpress. (**b**) A meta-analysis for BZW1 gene against clinical cohorts with lung cancer using PrognoScan. The data were performed using Cox proportional hazards regression analysis with 95% CI (confidence interval).

To identify the roles of the expression of BZW family members (BZW1 and BZW2) in cancer patients, we screened comprehensive datasets and found the corresponding hazard ratios and *p*-values from *in silico* analysis. The results showed that BZW1 had a more significant clinicopathological value than BZW2 in most cancer types. BZW1 was overexpressed in several cancer types and was associated with high hazard ratios in lung, pancreatic and colon cancer. BZW1 had especially significant *p*-values in lung cancer as determined via either microarray (Lung Meta-base, 6 cohorts, n = 1053, HR = 1.39), RNA-seq (The Cancer Genome Atlas, TCGA, n = 475, HR = 1.41), or both (Fig. 1a). Oppositely, BZW2 had a significant *p*-value in only glioma (Fig. S1). Therefore, we chose to further investigate the role of BZW1 in lung cancer.

To confirm the prognostic significance of BZW1 in lung cancer, we analyzed the hazard ratio and *Cox-p* value in microarray datasets from the Prognoscan website. Interestingly, we observed that BZW1 had a more powerful statistical value in lung cancer adenocarcinoma that in the squamous subtype (Fig. 1b).

### High BZW1 expression was associated with poor prognosis

**Figure 2:**
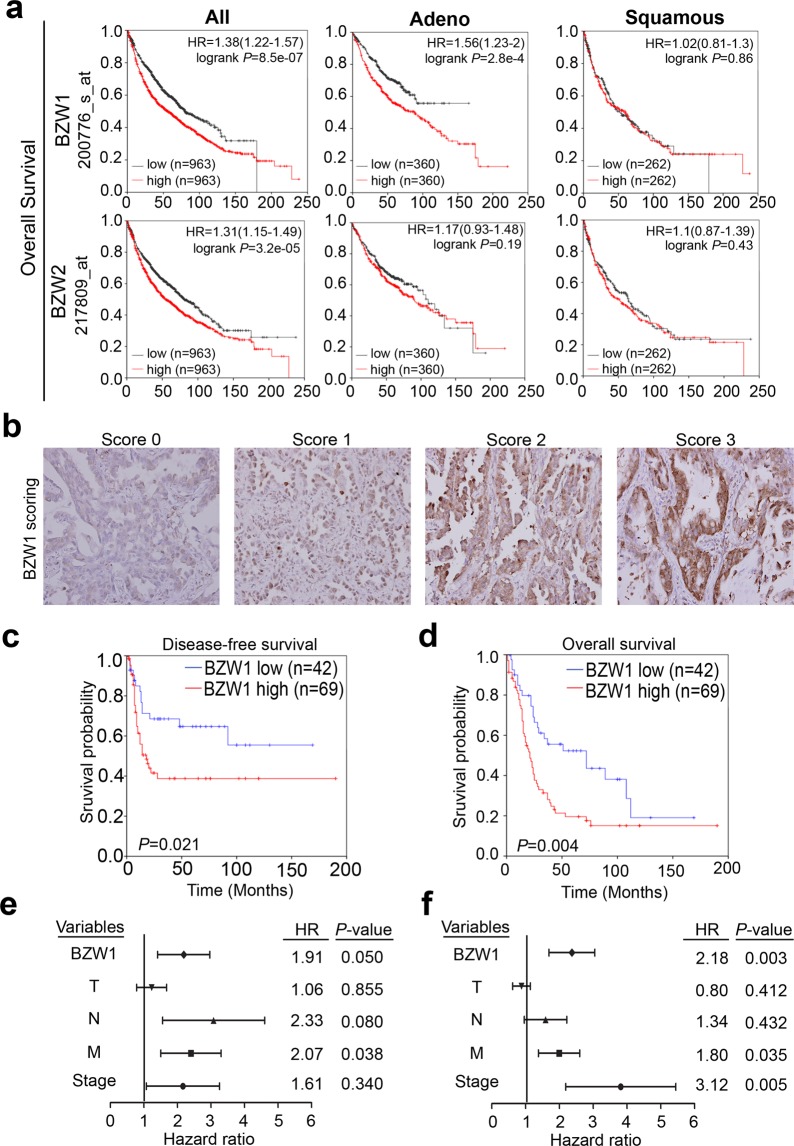
BZW1 is overexpressed in lung cancer and correlated with poor survival. (**a**) Kaplan-Meier analysis of *BZW1* and *BZW2* mRNA expression level at concurrently low or high levels or of others as determined by *in silico* datasets at the endpoint of overall survival in whole lung cancer patients, lung adenocarcinoma patients and lung squamous cell carcinoma patients, respectively. (**b**) Scores indicating BZW1 levels in representative lung tumor tissues from score 0~3. (**c,d**) Kaplan-Meier analysis of BZW1 protein expression at concurrently low or high levels or of others as determined by IHC staining at the endpoint of overall survival and disease-free survival probability in lung cancer patients (p = 0.021, p = 0.004, respectively). (**e**) Univariate Cox regression hazard ratio for risk of recurrence in patients with lung cancer. (**f**) Multivariate Cox regression hazard ratio for risk of death in patients with lung cancer.

We further analyzed how BZW1 expression correlated with patient survival times in clinical cohorts using the Kaplan-Meier plotter website (www.kmplotter.com). This population contains several microarray datasets combined with TCGA cohorts. We observed that high BZW1 (probe ID: 200766_s_at) expression levels correlated with poor overall survival (OS) and first progression (FP) (Figs 2a and S2). We further assessed patient cases based on histological classifications (adenocarcinoma and squamous cell carcinoma) in several datasets (Figs 2a and S3). The results revealed that BZW1 had a strong correlation with survival time in lung adenocarcinoma patients but not in patients with the squamous cell carcinoma subtype. In addition, BZW2 did not have a significant clinicopathological value in either adenocarcinoma or the squamous subtype (Fig. 2a). The data also showed trends that were consistent with those previously noted (Fig. 1b).

Next, we validated these findings by IHC staining for the BZW1 protein in clinical lung cancer tissues (Fig. 2b). The IHC results showed that the protein level of BZW1 in tumors was predominantly more elevated than that in the adjacent normal tissues. In comparison with low BZW1 expression (IHC scores 0–1), high BZW1 expression (IHC scores 2–3) was significantly correlated with poor OS and DFS probabilities (Fig. 2c,d). Univariate and multivariate analyses were performed for DFS probability (Fig. 2e,f). The univariate results revealed several clinicopathological parameters, including the BZW1 expression level and N/M status, that could be used for prognostic factors for patients with lung cancer (Tables 1 and S1). The multivariate analysis showed that the *p*-value of BZW1 was more significant than that of other genes. Thus, BZW1 has the potential to be an independent lung cancer prognostic factor (Table S2). These data also indicated that the upregulation of BZW1 might be closely associated with tumorigenesis and lung cancer progression.

**Table 1:** Univariate and multivariate analyses for BZW1 expression in lung cancer.

Variables	Comparison	HR (95% CI)	*P*-value
**Cox univariate analysis (OS)**			
BZW1	High vs. Low	2.02 (1.24–3.30)	0.005
T	T3-T4 vs. T1-T2	2.21 (1.39–3.52)	<0.001
N	N1-N3 vs. N0	3.29 (1.89–5.72)	<0.001
M	M1 vs. M0	3.19 (1.97–5.17)	<0.001
Stage	III, IV vs. I, II	4.22 (2.54–7.03)	<0.001
**Cox multivariate analysis (OS)**			
BZW1	High vs. Low	2.18 (1.30–3.63)	0.003
T	T3-T4 vs. T1-T2	0.80 (0.47–1.37)	0.412
N	N1-N3 vs. N0	1.34 (0.65–2.78)	0.432
M	M1 vs. M0	1.80 (1.04–3.13)	0.035
Stage	III, IV vs. I, II	3.12 (1.41–6.92)	0.005
**Cox univariate analysis (DFS)**			
BZW1	High vs. Low	2.04 (1.09–3.81)	0.026
T	T3-T4 vs. T1-T2	2.41 (1.35–4.30)	0.003
N	N1-N3 vs. N0	4.20 (1.94–9.08)	<0.001
M	M1 vs. M0	3.46 (1.93–3.21)	<0.001
Stage	III, IV vs. I, II	3.92 (2.01–7.65)	<0.001
**Cox multivariate analysis (DFS)**			
BZW1	High vs. Low	1.91 (1.00–3.65)	0.050
T	T3-T4 vs. T1-T2	1.06 (0.55–2.08)	0.855
N	N1-N3 vs. N0	2.33 (0.90–6.01)	0.080
M	M1 vs. M0	2.07 (1.04–4.10)	0.038
Stage	III, IV vs. I, II	1.61 (0.61–4.27)	0.340

### High BZW1 expression was observed in tumors and correlated with the metastatic status

**Figure 3:**
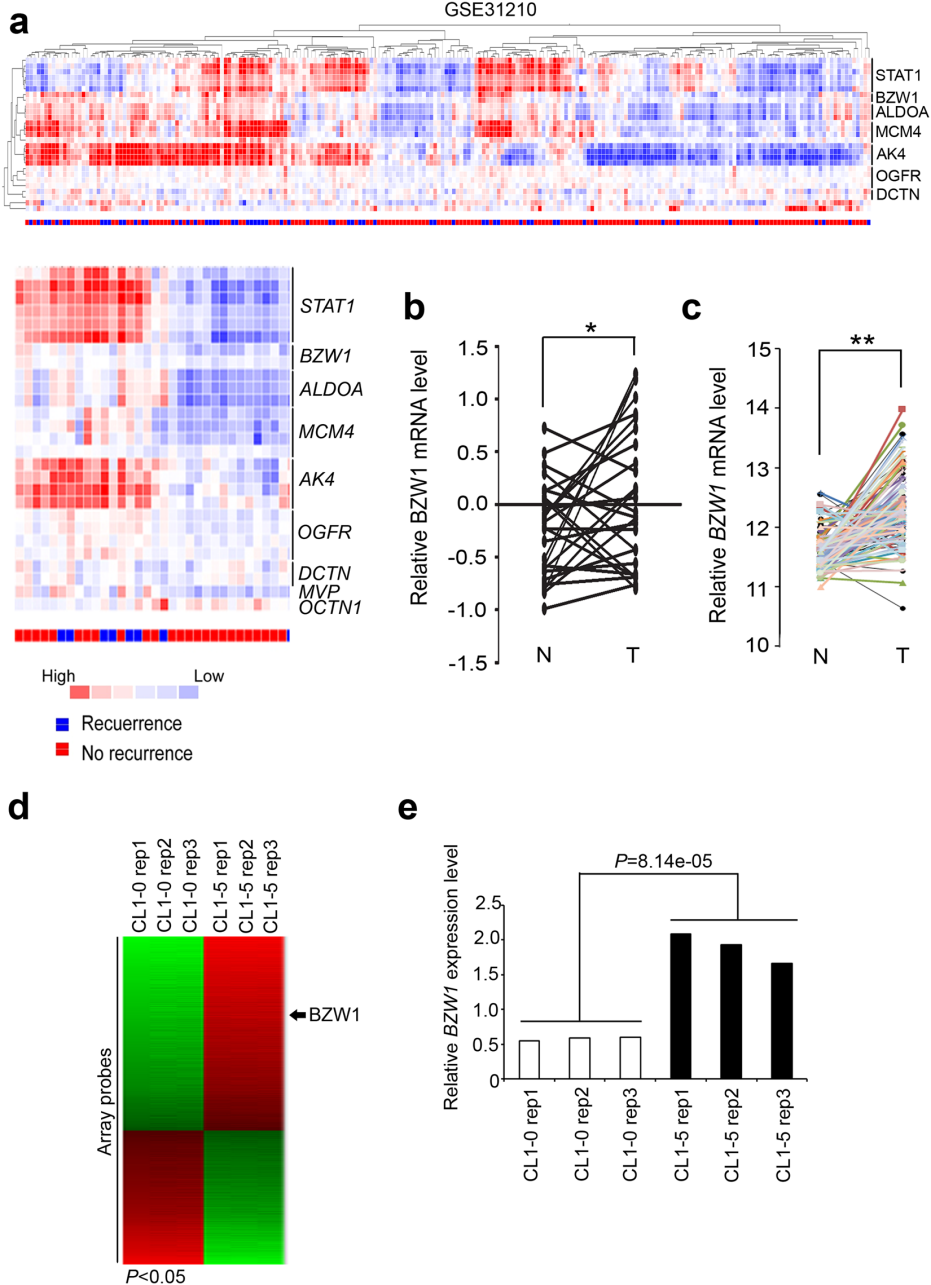
BZW1 was overexpressed in malignant part and correlated with recurrence status. (**a**) The heat-map involved the mRNA expression level of candidates’ genes, BZW1 correlated with clinical patient’s recurrence status by GSE31210. (**b,c**) BZW1 gene expression from the TCGA RNA sequencing database and the GEO microarray database (GSE7670) as identified by its corresponding probe in paired non-tumor and tumor tissues derived from lung cancer patients. (**d**) The heat-map involved the mRNA expression level of candidate genes in GSE42407 from CL1-5 compared CL1-0 with 1.5-fold change cut-off. (**e**) Box plot of mRNA level of BZW1 in CL1-5 part and its parental counterpart CL1-0 group by GSE42407 analyzed.

To evaluate the clinical pertinence of BZW1 in lung cancer patients, we analyzed the microarray database GSE31210. The heat map clearly showed that high BZW1 expression was associated with recurrence events in lung cancer patients (Fig. 3a). The signature revealed several candidate genes in this heat map, including BZW1, Aldolase A (ALDOA) and Adenosine Kinase 4 (AK4). Previously, we validated that the significant *p*-values of ALDOA and AK4 were indeed correlated with survival rates and the metastatic status^10,12^. Therefore, we proposed that BZW1 may be associated with lung cancer tumorigenesis or metastasis events. Moreover, the BZW1 levels in tumors obtained from clinical lung cancer cohorts were significantly higher than the levels in the adjacent normal tissues from GSE7670 (Fig. 3b). In addition, we observed consistency trends in the TCGA lung cancer cohort (Fig. 3c). We also performed microarray analysis of nonmetastatic CL1-0 and its counterpart cell line CL1-5 to identify genes critical for aberrant expression in lung cancer metastasis (Fig. 3d, GSE42407). Our data showed that BZW1, as one of the probes, was upregulated in CL1-5 cells compared to that in CL1-0 cells (Fig. 3e, *p* = 8.14e-05).

### BZW1 regulates the metastatic ability of lung cancer cells *in vitro*

**Figure 4:**
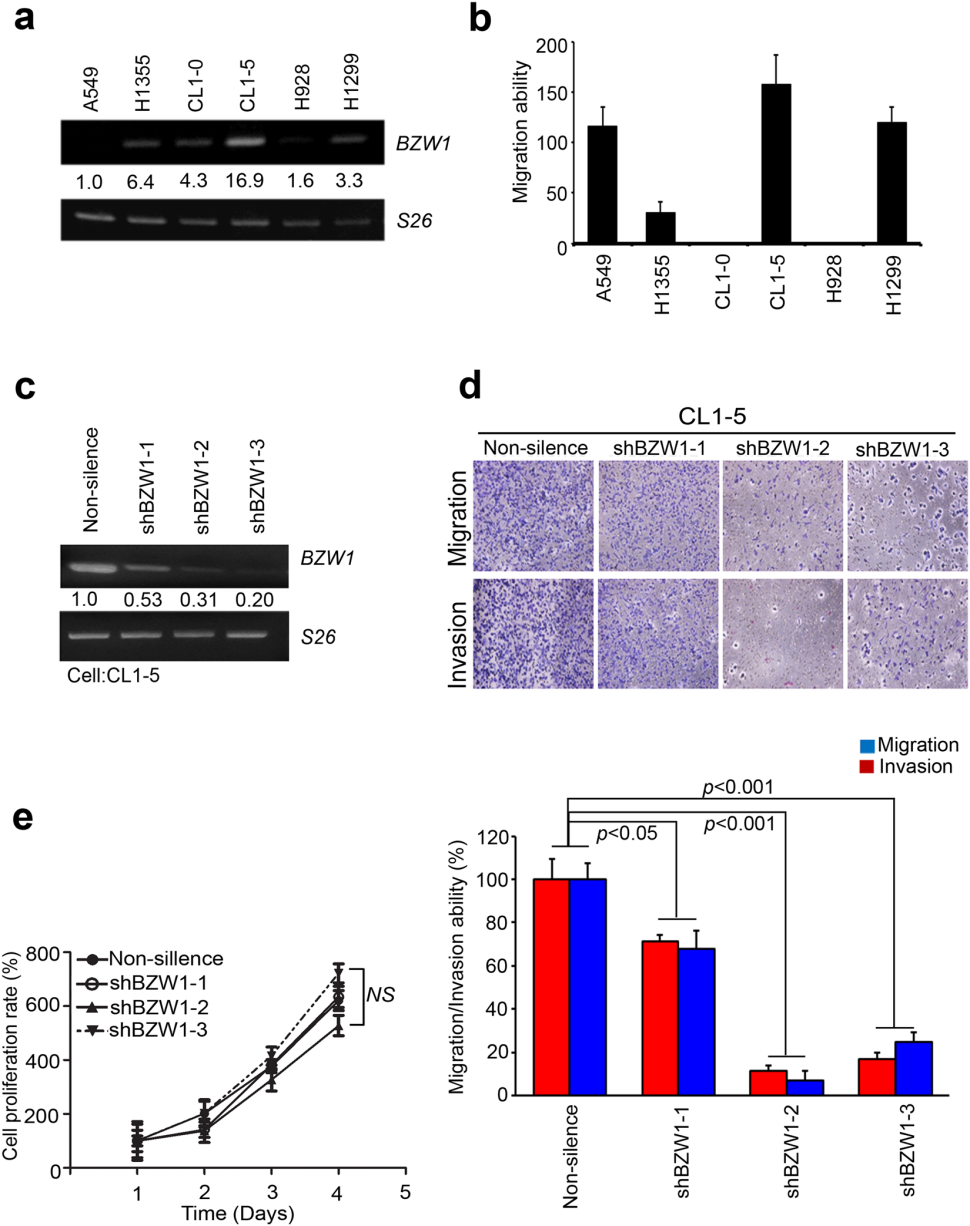
BZW1 promote metastasis and invasion phenotype in lung cancer cells. (**a**) Endogenous protein levels of BZW1 were analyzed on 6 lung cell lines was determined by western blotting on lung cell lines pattern. *S26* was used as internal control. (**b**) Quantification the migration ability on lung cell lines panel through boyden’s chamber assay. **(c**) RT-PCR assay of RNA level with or without BZW1 shRNA in CL1–5 cells. (**d**) Loss of BZW1 the metastasis and invasive abilities of lung cancer cells. Invasive abilities of CL1–5 and quantification image expressing BZW1 three independent shRNA. (**e**) Cell proliferation rate by MTT assay with or without BZW1 shRNA in CL1–5 cell model.

To confirm whether BZW1 indeed affected metastatic events in lung cancer, we detected the endogenous expression of BZW1 in lung cancer cell panels (Fig. 4a). We also evaluated the metastatic ability of each lung cancer cell line using the Boyden’s chamber assay (Fig. 4b). Merging the evidence, we noted a positive correlation between endogenous BZW1 expression and cellular invasive/migratory activity in various lung cancer cell lines. As shown in Fig. 4a, BZW1 was plenteously expressed in metastatic lung cancer cell lines, e.g., CL1-5, but its expression was relatively lower in nonmetastatic lung cancer cell lines, e.g., CL1-0 and H928. Further, the results matched the mRNA levels of CL1-0 and CL1-5 in GSE42407 (Fig. 3e). The knockdown of BZW1 by its specific shRNA significantly mediated the invasive/migratory capabilities of the metastatic lung cancer cell line CL1-5 (Fig. 4c,d). These results implicated that BZW1 expression is causally associated with cellular invasive abilities *in vitro* regardless of the cell proliferation capability of lung cancer cells (Fig. 4e).

### BZW1 expression was associated with the EGFR in lung cancer

**Figure 5:**
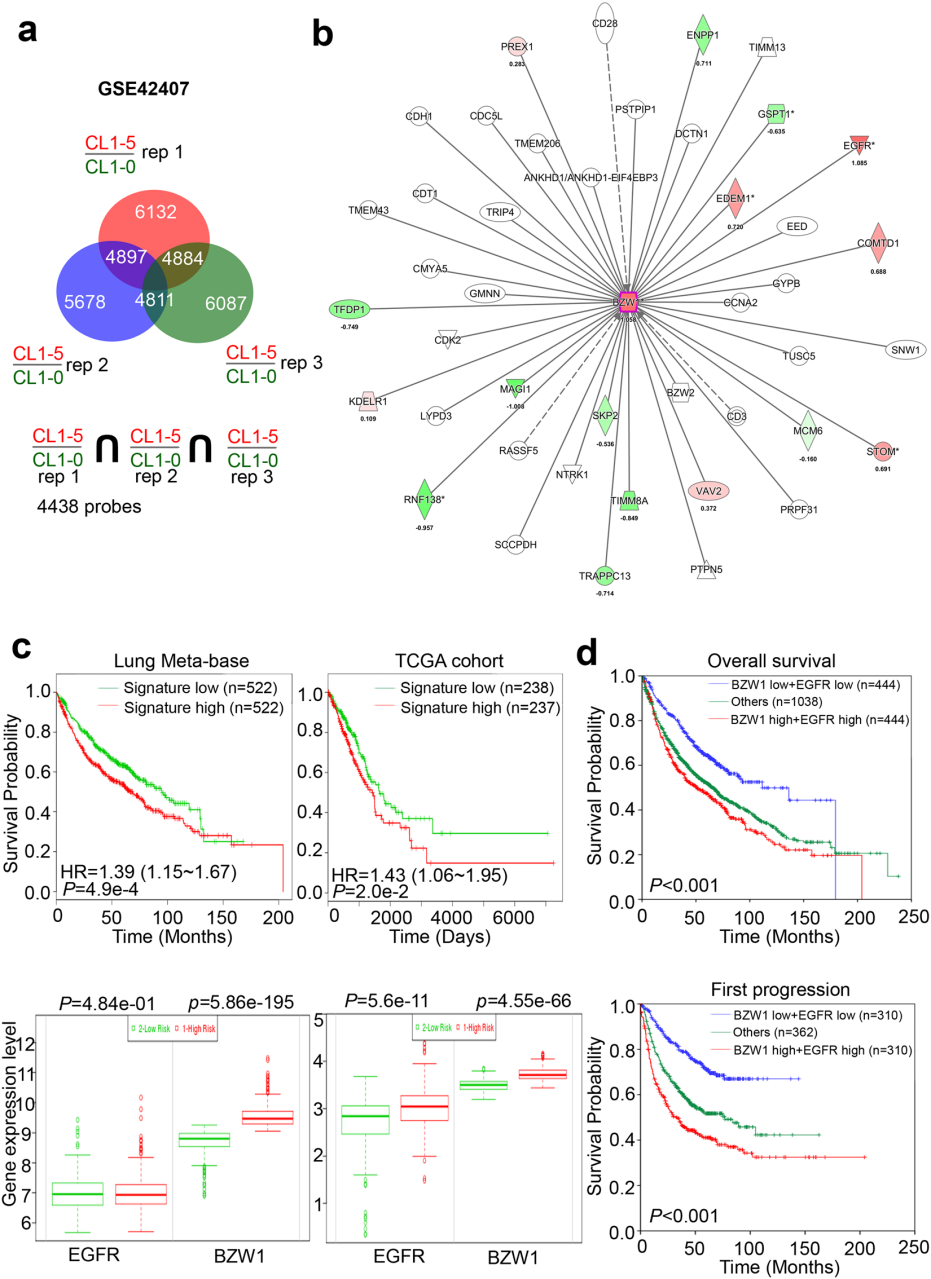
Overexpression of BZW1 and EGFR is a strong poor prognosis marker for lung cancer patients. (**a**) Microarray analysis showed which gene activation/inhibition profiles in CL1-5 or CL1-0 cells. The genes were recruited with 1.5-fold change at least. (**b**) The network was being predicted based on the BZW1-based signature that in the Ingenuity IPA database overlaid with microarray data from CL1-5 cells versus CL1-0 cells with a 1.5-fold change cut-off. The intensity of the node color indicates the degree of upregulate-(red) and downregulate (green) regulation following lung metastatic interatomics. (**c**) Kaplan–Meier plot analyzed by BZW1 with its interaction molecules in lung cancer from microarray and TCGA cohort, respectively. Stratified by three group (BZW1 high plus EGFR high, BZW1 low with EGFR low and others, respectively) (**d**) Kaplan–Meier plot analyzed of overall and fist progression by BZW1 with its interaction molecules in lung cancer, respectively. Stratified by three group (BZW1 high plus EGFR high, BZW1 low with EGFR low and others, respectively).

Previously reported, the CL1-5 cells was generated through artificially selected from its counterpart CL1-0 cells. Therefore, CL1-5 had more migration/invasion ability. Due to our data indicates BZW1 had closely correlation between expression levels with metastatic ability in lung cancer cells. Therefore, we regard the CL1-0 cells as the benign cell and CL1-5 has the malignant cell of our panel. We further performed microarray analysis from GSE42407. Normalized data and predicted potential molecules were activated in CL1-5 cells by genespring and ingenuity pathway analysis (IPA), respectively. The results focused on the BZW1-based signature by inputting candidate probes exceeding the 1.5-fold change cutoff in CL1-5 cells compared with that in CL1-0 cells (Fig. 5a). We found that BZW1 was upregulated in CL1-5 cells compared with that in CL1-0 cells (Fig. 5b). It has been reported that CL1-0/CL1-5 has EGFR wild-type and CL1-5 with EGFR expression levels higher than CL1-0. In addition, the signature also revealed several targets that may be upregulated/downregulated by BZW1 induction. EGFR was one target who mRNA levels were increased in CL1-5 cells (Fig. 5b). Therefore, we integrated the mRNA levels of BZW1 and EGFR with the survival curves of online lung cancer databases. The combination of markers was associated with poor survival, and this association was increased in the high risk group (Fig. 5c). High BZW1 expression combined with high EGFR RNA levels were significantly correlated with worse patient overall survival and first progression survival (Fig. 5d). Combining all evidence, we established a relationship between BZW1 and EGFR in lung cancer metastasis.

## Discussion

In all eukaryotes, Basic Leucine Zipper (bZIP) family regulate critical transcription processes^13^. In plants, bZIPs are predominant regulators of many physiological and developmental processes, including biotic stress responses, morphogenesis and seed formation^14–16^. In humans, the bZIP family is the second-largest dimerizing transcription factor family. These proteins are encoded by 51 genes^13^. In many human cancers, a number of oncogenic transcription factors, such as the bHLH and bZIP families, are overactive in regulating oncoproteins that play critical roles in upstream signaling pathways^17^. Inhibition of these transcription factors is a novel target of cancer therapeutic strategies. Thus far, using dominant-negative peptides, the phenomenon of dimerization of the bHLH and bZIP families has been disrupted. These synthetic peptides lack DNA-binding domains and can therefore interrupt endogenous TF dimerization and DNA binding^18^. Recently, small molecules in development were reported to disrupt protein-protein interactions. These small molecules were considered more drug-like than the peptides^19^. Some reports showed that cancer cell growth was inhibited after the interruption of c-Myc and STAT3 dimerization by small molecules^20,21^. However, some side effects are associated with this type of therapeutic strategy because the surfaces of transcription factors are too large for binding small molecules. Thus, other novel strategies for disrupting transcription factor dimerization are needed to cure cancer.

The protein structures of Basic Leucine Zipper and W2 domain-containing protein 1 (BZW1, BZAP45) contain a leucine zippers and a W2 domains, respectively^22,23^. The family members include BZW1 and a paralog gene, BZW2^24,25^. Focusing on domain structure, BZW1/BZW2 compose an N-terminal basic leucine zipper fragment and a C-terminal nucleotide binding motif. bZIP has been regarded as the coregulator for several transcription factors. Based on a previous report, BZW1 could enhance the transcription activity of histone H4 to control the cell cycle. Similarly, there is evidence that BZW2 regulates the G2/M cell cycle transition and is involved in the Akt/mTOR signaling pathway in osteosarcoma^26^. However, more studies pointed out that BZW1/BZW2 dysfunction in tumorigenesis depends on an unknown pathway. Therefore, we screened BZW1 and BZW2 at the RNA level in several cancer types^27^. The results showed that BZW1 plays a significant role in cancers of the lung, pancreas, colon, head and neck, and prostate; as such, its potential clinical value and involved molecular mechanisms still need to be further investigated. Conversely, BZW2 lacks suitable probes and patient cohorts to evaluate its clinical value. Owing to the number of patients, our cohort is not large enough, and it is biased towards early stage cancer, which can easily lead to significant differences. We are recruiting more patients and establishing a validation cohort to verify BZW1 as a prognostic marker in lung cancer.

One of the most powerful strategies for determining the role of BZW1 in tumorigenesis is exploring the consequences of protein-protein interaction (PPIs). Previously, BZW1 was identified to form a complex with several factors, including human pleiotropic regulator 1 (PLRG1) and spliceosome proteins cell division cycle 5-like (CDC5L), via protein-protein interactions^28,29^. In addition, large-scale mapping by mass spectrometry showed that BZW1 is available in macromolecular complexes that directly bind with dynactin1 (DCTN1)^30,31^. Moreover, BZW1 may interact with BZW2 in the resulting network (BioPlex)^32,33^. Here, we provided several indications that BZW1 can coordinate with EGFR in lung tumorigenesis^34^. EGFR has always served as an important marker for patients with lung cancer^35,36^. Chemotherapy or other therapies rely on its mutation status^37^. However, details about the mechanism by which BZW1 regulates EGFR activity in cancer still needs further investigation.

In our data, we provided evidence that BZW1 expression levels correlated with recurrence events in a lung cancer cohort. We constructed a heat map relating gene probe expression levels with recurrence events in GSE31210 by hierarchical clustering. The data showed that BZW1 had trends consistent with those of *ALDOA*, *AK4* and *DCTN*. ALDOA and AK4 have been identified to be correlated with the metastatic activity of lung cancer cells^12^. ALDOA stabilizes the HIF1α protein and forms a positive feedback pathway to promote lung cancer metastasis. In addition, AK4 suppresses the regulator ATF3 to regulate lung tumorigenesis. Hence, we observed that BZW1 and its binding molecule DCTN show similar trends in lung cancer. Going forward, we should explore the detailed molecular mechanisms induced by BZW1.

Cancer hallmarks include evading apoptosis, self-sufficient growth signals, sustained angiogenesis, insensitivity to antigrowth signals and limitless replication potential. In recent research, scientists used cancer hallmark-based gene signatures to predict the prognoses of patients with cancer^38–41^. The predictive accuracy of using cancer hallmark-based gene signatures was higher than that achieved without using systematically selected genes. Further, accurate prediction of cancer prognosis will be valuable for precise diagnosis and personalized treatment strategies. We investigated the correlation of BZW1 with tissue invasion and metastasis in a cell line model and clinical events of lung cancer. We found that BZW1 expression is an independent prognostic factor and has great prognostic significance for both OS and DFS in lung cancer patients.

In conclusion, our current study revealed that BZW1 has a more significant clinicopathological value than BZW2 in most cancer types. Further, high BZW1 expression predicts poor prognosis better in lung adenocarcinoma subtype patients than in squamous subtype carcinoma patients. BZW1 expression is associated with the EGFR mutant type in lung cancer, and knockdown of BZW1 expression significantly decreases the cellular migration ability *in vitro*. Developing inhibitors for BZW1 PPIs may be a potential therapeutic strategy for lung adenocarcinoma.

## Supplementary information

Supplementary information
