# Supplementary material for: Overexpression of BZW1 is an independent poor prognosis marker and its down-regulation suppresses lung adenocarcinoma metastasis
Source: Sci Rep. 2019 Oct 10;9:14624. doi: 10.1038/s41598-019-50874-x (PMC6786993; doi:10.1038/s41598-019-50874-x)

Supplement information

Overexpression of BZW1 is an independent poor prognosis marker and its down-regulation suppresses lung adenocarcinoma metastasis

Jean Chiou[1*], Yu-Chan Chang[1*], Yi-Hua Jan[1], Hsing-Fang Tsai[1], Chih-Jen Yang[2], Ming-Shyan Huang[3], Yung-Luen Yu[4-7#] and Michael Hsiao[1,8#]

1. Genomics Research Center, Academia Sinica, Taipei, Taiwan
2. Department of Internal Medicine, Kaohsiung Municipal Ta-Tung Hospital, Kaohsiung Medical University Hospital, Kaohsiung Medical University, Kaohsiung Taiwan
3. Department of Internal Medicine, E-DA Cancer Hospital, School of Medicine, I-Shou University, Kaohsiung, Taiwan
4. The PhD. Program for Cancer Biology and Drug Discovery, China Medical University and Academia Sinica, Taichung, Taiwan
5. Graduate Institute of Biomedical Science, China Medical University, Taichung, Taiwan
6. Center for Molecular Medicine, China Medical University Hospital, Taichung, Taiwan
7. Department of Biotechnology, Asia University, Taichung, Taiwan
8. Department of Biochemistry, College of Medicine, Kaohsiung Medical University, Kaohsiung, Taiwan

Table S1. Clinical relevance of BZW1 expression in lung cancer

		BZW1 expression, n (%)		
Characteristics	n	Low (n =42)	High (n =69)	*P* value
Age				
＜65y	63	26(41.3)	37(58.7)	0.393
≧65y	48	16(33.3)	32(66.7)	
Sex				
Male	61	23(37.7)	38(62.3)	0.975
Female	50	19(38.0)	31(62.0)	
Smoking status				
No	45	21(46.7)	24(53.3)	0.113
Yes	66	21(31.8)	45(68.2)	
Histological type				
Adenocarcinoma	68	29(42.6)	39(57.4)	0.216
Squamous carcinoma	35	12(34.3)	23(65.7)	
Large cell carcinoma	8	1(12.5)	7(87.5)	
Stage[#]				
I+ II	45	19(42.2)	26(57.8)	0.432
III+ IV	66	23(34.8)	43(65.2)	
Tumor status				
T1+ T2	74	31(41.9)	43(58.1)	0.213
T3+ T4	37	11(29.7)	26(70.3)	
Lymph node status				
N0	37	17(45.9)	20(54.1)	0.222
N1-3	74	25(33.8)	49(66.2)	
Distal metastasis status				
M0	78	32(41.0)	46(59.0)	0.287
M1	33	10(30.3)	23(69.7)	
Recurrence status				
No	64	22(34.4)	42(65.6)	0.380
Yes	47	20(42.6)	27(57.4)	

*p value<0.05 was considered statistically significant (Student's t-test for continuous variables and Pearson's chi-square test for variables). SD represents the standard deviation. [#]The tumor stage, tumor, lymph node, and distal metastasis status were classified according to the international system for staging lung cancer.

Table S2. Univariate and multivariate analysis for BZW1, ALDOA, AK4, and stage in lung cancer patients.

Variables		OS		DFS	
		HR (95% CI)	*P*	HR(95% CI)	*P*
**Cox univariate analysis**					
BZW1 expression	High vs. low	0.66 (0.38-1.14)	0.134	1.64 (1.31-2.31)	0.022*
ALDOA expression	High vs. low	1.34 (0.82-2.19)	0.236	1.89 (1.00-3.57)	0.05*
AK4 expression	High vs. low	1.52 (0.91-2.54)	0.109	1.98 (1.02-3.81)	0.042*
T stage	T3-4 vs. T1-2	2.13 (1.16-3.94)	0.015	1.38 (0.67-2.85)	0.382
N stage	N1-3 vs. N0	3.21 (1.84-5.61)	<0.001*	3.87 (1.79-8.34)	<0.001*
M stage	M1 vs. M0	3.13 (1.85-5.28)	<0.001*	2.63 (1.39-4.99)	0.003*
**Cox multivariate analysis**					
BZW1 expression	High vs. low	1.50 (1.28-1.90)	0.021*	1.46 (1.22-1.92)	0.042*
ALDOA expression	High vs. low	1.15 (0.67-1.98)	0.604	1.82(0.88-3.77)	0.105
AK4 expression	High vs. low	1.12 (0.62-2.03)	0.704	1.17 (0.54-2.52)	0.692
T stage	T3-4 vs. T1-2	1.44 (0.75-3.80)	0.276	1.74(0.70-4.33)	0.237
N stage	N1-3 vs. N0	2.97 (1.62-5.47)	<0.001*	3.82(1.68-8.72)	<0.001*
M stage	M1 vs. M0	1.99 (1.06-3.74)	0.033	1.29(0.53-3.12)	0.575

Table S3. The correlations between IHC expression level of BZW1, ALDOA, and AK4

Correlation					
Spearman's rho			BZW1	ALDOA	AK4
	BZW1	Correlation	1.000	0.131	0.206
		P-value	.	0.223	0.054
		n	88	88	88
	ALDOA	Correlation	0.131	1.000	0.298
		P-value	0.223	.	0.005
		n	88	88	88
	AK4	Correlation	0.206	0.298	1.000
		P-value	0.054	0.005	.
		n	88	88	88

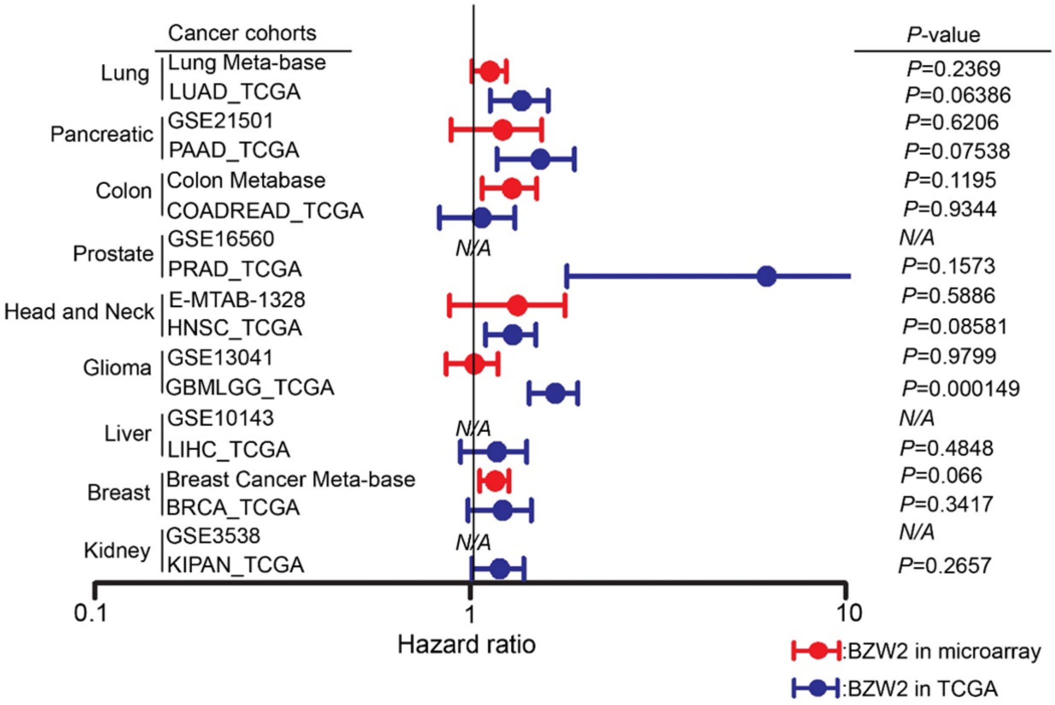

Figure S1. A meta-analysis for BZW1 gene against clinical cohorts with multiple cancer includes microarray and TCGA cohort by using Survexpress.

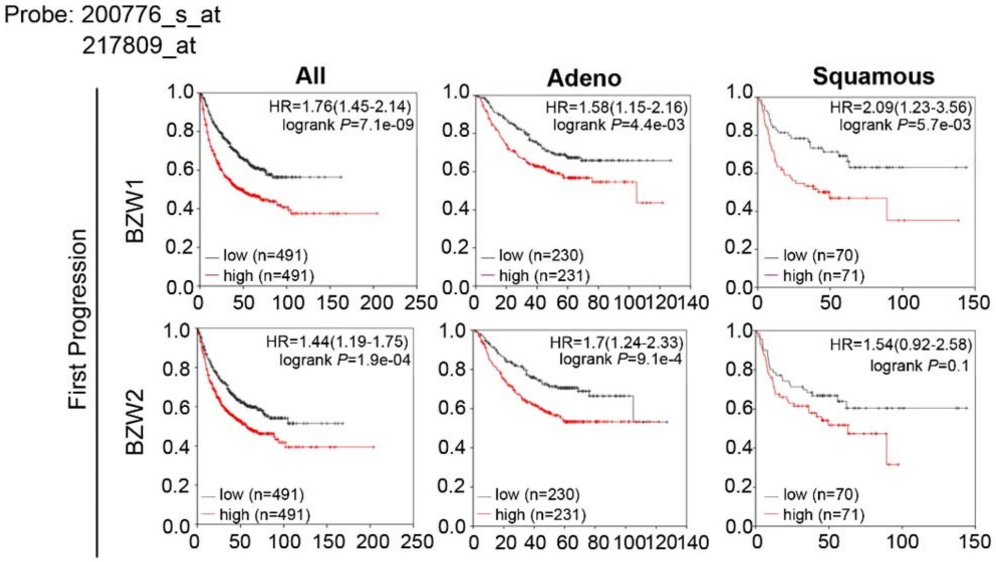

Figure S2. Kaplan-Meier analysis of BZW1and BZW2 mRNA expression level at concurrently low or high levels or of others as determined by *in silico* datasets at the endpoint of first progression in whole lung cancer patients, lung adenocarcinoma patients and lung squamous cell carcinoma patients, respectively.

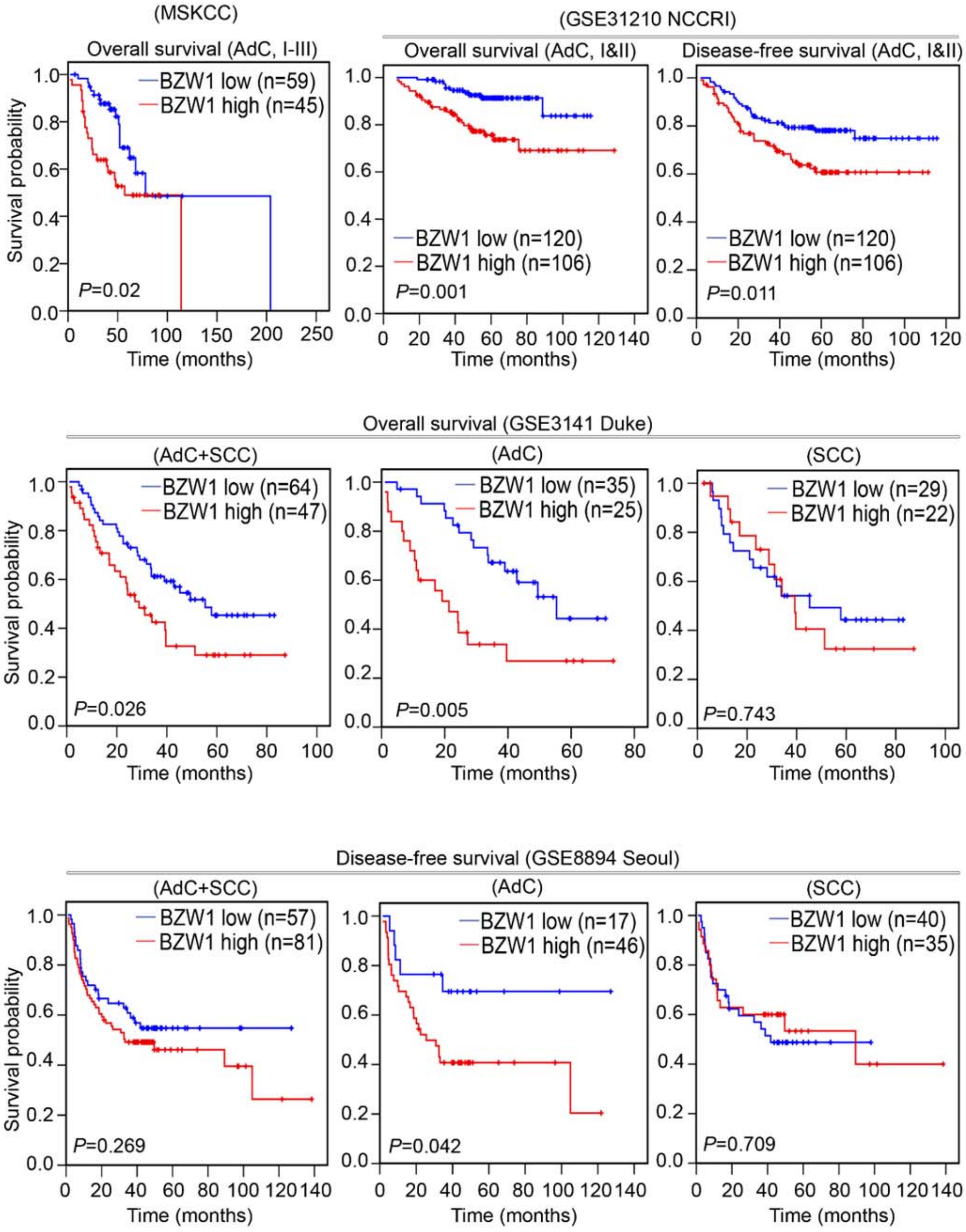

Figure S3. Kaplan-Meier analysis of BZW1 mRNA expression level at concurrently low or high levels or of others as determined by *in silico* datasets at the endpoint of overall survival in whole lung cancer patients, lung adenocarcinoma patients and lung squamous cell carcinoma patients, respectively.

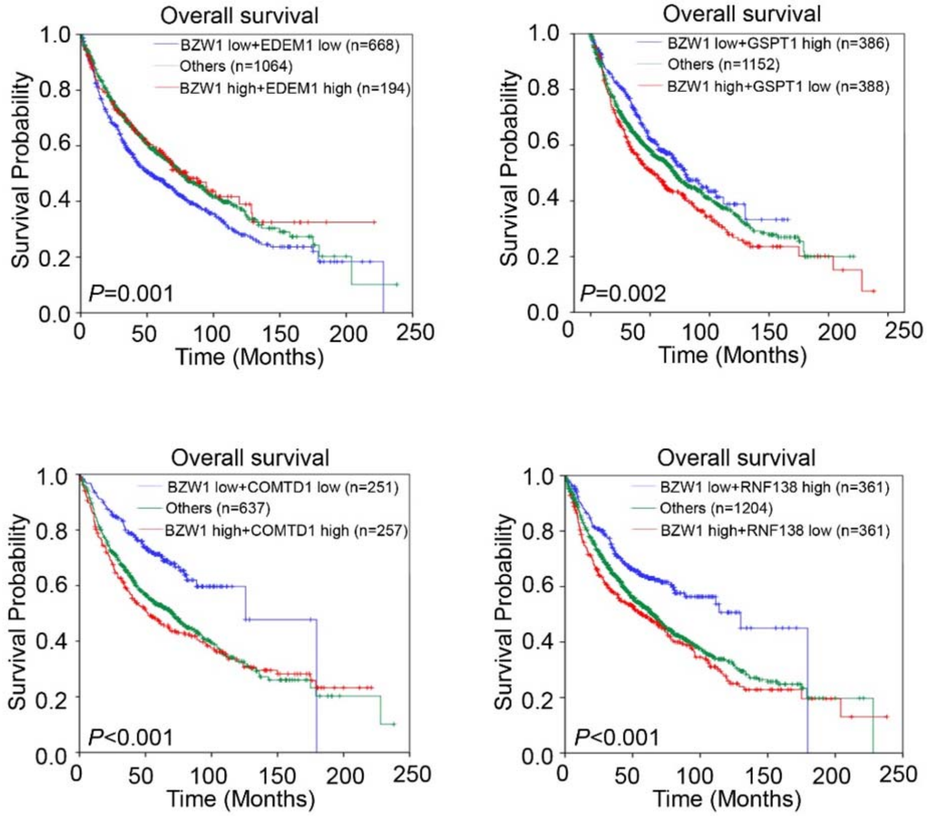

Figure S4. Kaplan–Meier plot of overall survival by BZW1 with its interaction molecules in lung cancer. Stratified by three group (BZW1 high plus EDEM1/COMTD1 high, BZW1 low with EDEM1/COMTD1 low and others, respectively) or (BZW1 high plus GSPT1/RNF138 low, BZW1 low with GSPT1/RNF138 high and others, respectively)

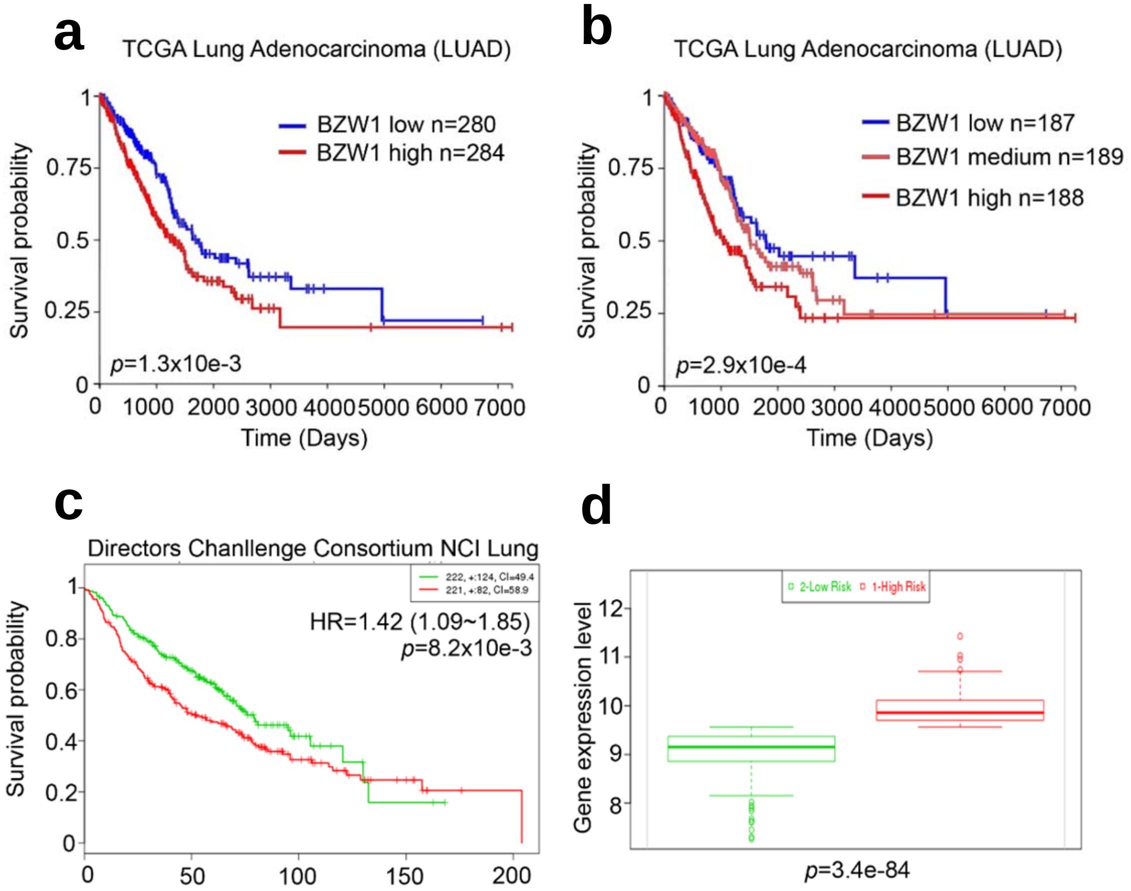

Figure S5. Kaplan–Meier plot of overall survival by BZW1 mRNA expression level at concurrently low or high levels or of others as determined by *in silico* datasets at the endpoint of overall survival in lung adenocarcinoma patients. (a) and (b): Kaplan–Meier plot of overall survival in TCGA(LUAD) cohorts through two grouped and three grouped. The data showed BZW1 remain as an significant prognostic factor in lung cancer patients. (c) and (d): GSE30219(Rousseaux) cohort also revealed the consistent result.

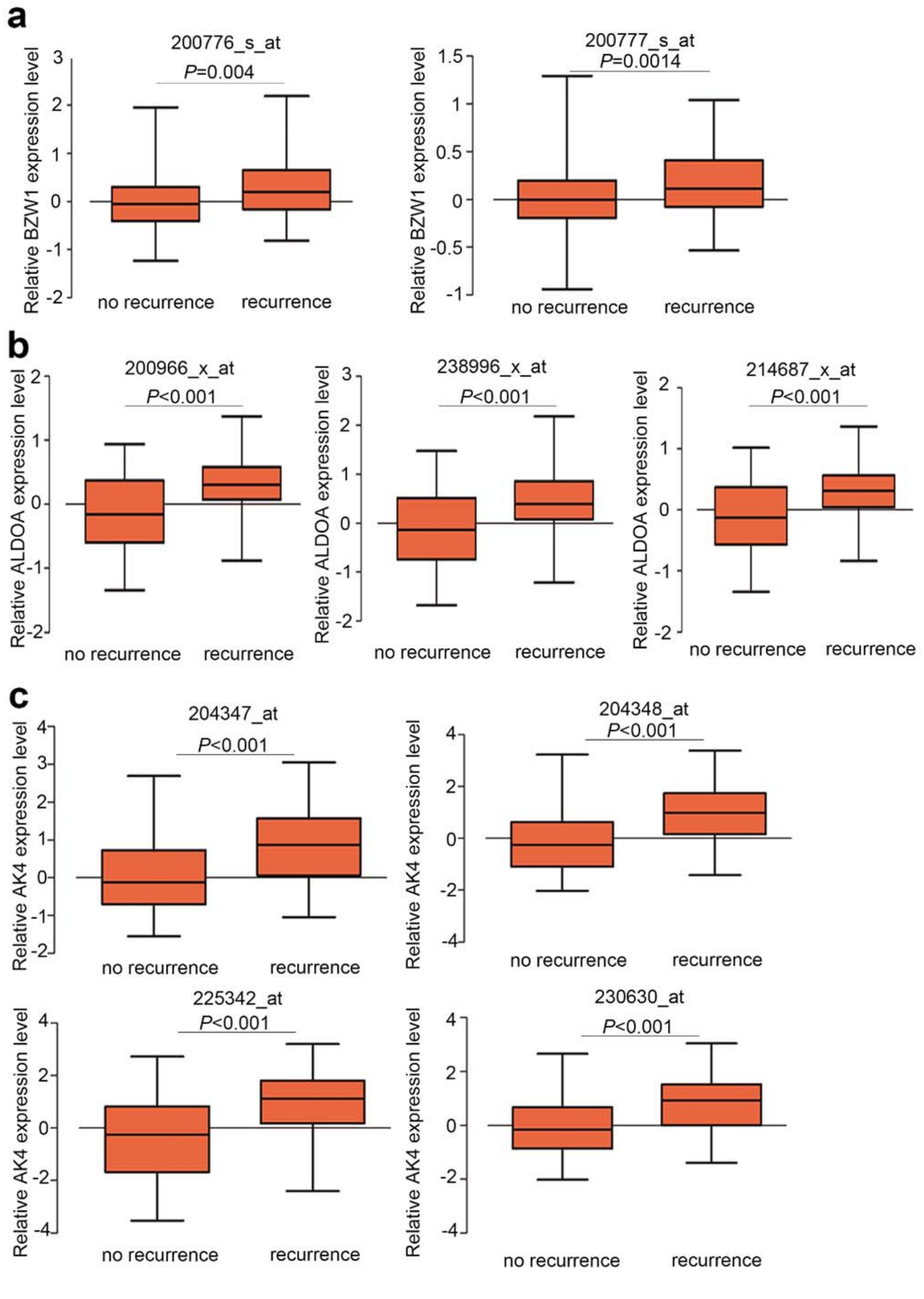

Figure S6. mRNA expression profiles in patients with recurrence in GSE31210 dataset. (a) BZW1 mRNA expression levels were higher in recurrent patients. *P*-value was obtained with t-test. (b) ALDOA mRNA expression levels were higher in recurrent patients. *P*-value was obtained with t-test. (c) AK4 mRNA expression levels were higher in recurrent patients. *P*-value was obtained with t-test.

Original Raw Images Information

Figure 4(a): Full length RT-PCR Agarose gel images taken by Canon Powershot G12 Digital Camara
Figure 4(c): Full length RT-PCR Agarose gel images taken by Canon Powershot G12 Digital Camara

Figure 4 (a)

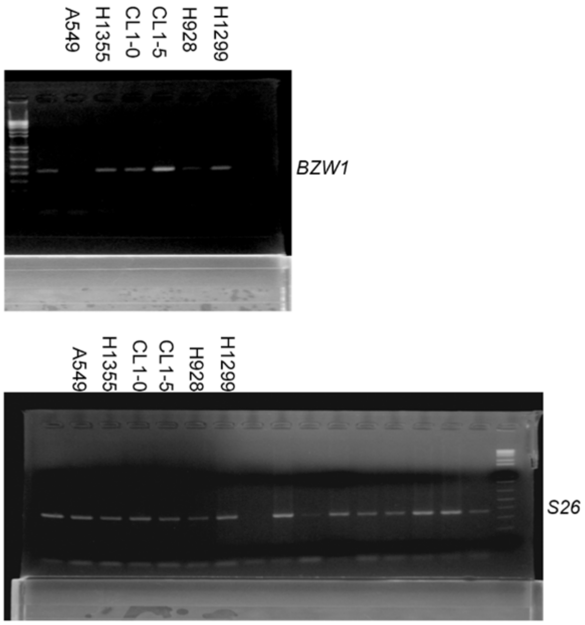

Figure 4(c)

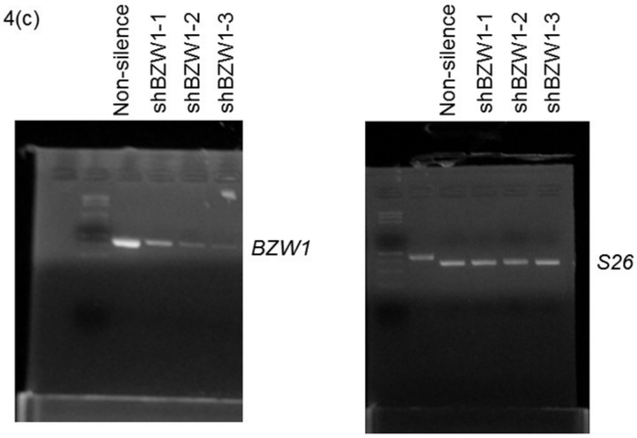

Supplement: Supplementary file 1 — Supplementary information [file 41598_2019_50874_MOESM1_ESM.pdf]
